# Polymer-mineral scaffold augments in vivo equine multipotent stromal cell osteogenesis

**DOI:** 10.1186/s13287-018-0790-8

**Published:** 2018-03-09

**Authors:** Wei Duan, Cong Chen, Masudul Haque, Daniel Hayes, Mandi J. Lopez

**Affiliations:** 1 Laboratory for Equine and Comparative Orthopedic Research, Louisiana State University, Baton Rouge, LA USA; 2 Department of Biomedical Engineering, Pennsylvania State University, University Park, PA USA

**Keywords:** Bone, Adipose, Bioreactor, Murine, Computed tomography, Microstructure

## Abstract

**Background:**

Use of bioscaffolds to direct osteogenic differentiation of adult multipotent stromal cells (MSCs) without exogenous proteins is a contemporary approach to bone regeneration. Identification of in vivo osteogenic contributions of exogenous MSCs on bioscaffolds after long-term implantation is vital to understanding cell persistence and effect duration.

**Methods:**

This study was designed to quantify in vivo equine MSC osteogenesis on synthetic polymer scaffolds with distinct mineral combinations 9 weeks after implantation in a murine model. Cryopreserved, passage (P)1, equine bone marrow-derived MSCs (BMSC) and adipose tissue-derived MSCs (ASC) were culture expanded to P3 and immunophenotyped with flow cytometry. They were then loaded by spinner flask on to scaffolds composed of tricalcium phosphate (TCP)/hydroxyapatite (HA) (40:60; HT), polyethylene glycol (PEG)/poly-l-lactic acid (PLLA) (60:40; GA), or PEG/PLLA/TCP/HA (36:24:24:16; GT). Scaffolds with and without cells were maintained in static culture for up to 21 days or implanted subcutaneously in athymic mice that were radiographed every 3 weeks up to 9 weeks. In vitro cell viability and proliferation were determined. Explant composition (double-stranded (ds)DNA, collagen, sulfated glycosaminoglycan (sGAG), protein), equine and murine osteogenic target gene expression, microcomputed tomography (μCT) mineralization, and light microscopic structure were assessed.

**Results:**

The ASC and BMSC number increased significantly in HT constructs between 7 and 21 days of culture, and BMSCs increased similarly in GT constructs. Radiographic opacity increased with time in GT-BMSC constructs. Extracellular matrix (ECM) components and dsDNA increased significantly in GT compared to HT constructs. Equine and murine osteogenic gene expression was highest in BMSC constructs with mineral-containing scaffolds. The HT constructs with either cell type had the highest mineral deposition based on μCT. Regardless of composition, scaffolds with cells had more ECM than those without, and osteoid was apparent in all BMSC constructs.

**Conclusions:**

In this study, both exogenous and host MSCs appear to contribute to in vivo osteogenesis. Addition of mineral to polymer scaffolds enhances equine MSC osteogenesis over polymer alone, but pure mineral scaffold provides superior osteogenic support. These results emphasize the need for bioscaffolds that provide customized osteogenic direction of both exo- and endogenous MSCs for the best regenerative potential.

## Background

A viable graft composed of adult multipotent stromal cells (MSCs) and biocompatible scaffold is a promising approach to enhance natural bone formation and augment current treatment strategies for equine traumatic bone injury [1, 2]. Autologous autograft promotes bone formation by local cells, but harvest morbidity in addition to variable cell survival and graft quantity and quality help fuel the search for regenerative medicine alternatives [3, 4]. Osteogenic capabilities of equine bone marrow-derived MSCs (BMSCs) and adipose-derived MSCs (ASCs) vary with culture conditions and scaffold carriers [5–8]. Given the inherent responsiveness of undifferentiated cells to their surroundings [1, 4, 9], it is vital to confirm in-vitro MSC characteristics in vivo. This is especially true since cell-based therapies are designed to enhance bone healing in potentially unfavorable conditions.

The natural bone fracture environment has over 200 noncollagenous matrix proteins and collagen and hydroxyapatite (HA) nanostructure that guides native progenitor cell osteogenesis [9]. Through structure and composition, functional scaffolds create a biomimetic environment for endo- and exogenous cells [9–14]. Mineral-based scaffolds have good biomimetic characteristics, but brittle mechanical properties complicate implant customization, surgical stabilization, and biological incorporation [15]. Scaffolds composed of both synthetic polymers and minerals such as HA and tricalcium phosphate (TCP) have biomimetic characteristics of inorganic matrix but greater flexibility [15, 16]. A scaffold composition that supports consistent, predictable tissue formation by MSCs from distinct tissues and donors is appealing for clinical application.

Poly-l-lactic acid (PLLA) and polyethylene glycol (PEG) are biocompatible, nontoxic polyester polymers [17, 18]. Distinct PLLA:PEG ratios have predictable hydrolytic degradation [19–23]. Addition of HA to PLLA scaffolds enhances murine osteoblast cell line protein adsorption, suppresses cell apoptosis, and enhances survival, growth, and osteogenic gene expression [24, 25]. Composite PLLA/TCP scaffold increases endogenous alkaline phosphatase (ALP) activity in human ASCs over PLLA alone [14]. Information about equine MSC-scaffold osteogenesis is relatively limited [26], but this contemporary knowledge supports a potential for polymer-mineral scaffold carriers to support the process [20].

Homogenous distribution of viable MSCs throughout scaffold carriers is crucial for organized tissue matrix production [27]. Bioreactor dynamic cell loading under optimal conditions limits cell loss and ensures distribution [28] important for neovascularization and de novo tissue formation [29]. Spinner flask bioreactors limit cell cluster formation, facilitate uniform cell seeding, and support nutrient and waste exchange [30, 31]. Additionally, exposure to fluid flow for as little as 15 min upregulates murine MSC osteogenic gene expression [32, 33]. Hence, spinner flasks provide an efficient mechanism for consistent construct loading that may help drive osteoblastic differentiation and maturation.

Given the potential of equine ASCs and BMSCs to augment bone generation [34, 35], targeted work is necessary to identify polymer-mineral bioscaffold carriers [36, 37] that reliably promote equine MSC osteogenesis in vivo. This study was designed to quantify osteogenesis by equine ASCs and BMSCs on scaffold carriers composed of TCP/HA (40:60; HT; Scaffdex™ Ltd., Tampere, Finland), PEG/PLLA (60:40; GA) and PEG/PLLA/TCP/HA (36:24:24:16; GT) in an athymic mouse model. The tested hypothesis was that osteogenesis is comparable on GA and GT scaffolds with either ASCs or BMSCs and greater than on HT scaffolds with either cell type. It was further hypothesized that the same scaffolds with MSCs have greater osteogenesis than those without.

## Methods

### Study design

Cell passage (P)1 cryopreserved equine BMSCs and ASCs were culture expanded to P3. Cells were loaded onto one commercially available mineral (HT) and two novel polymer-mineral scaffolds (GA and GT) via a spinner flask bioreactor. Passage 3  immunophenotype and cell viability on scaffolds after 7 and 21 days of static culture were determined. Scaffolds with and without cells were implanted subcutaneously in athymic mice. Radiographs were performed immediately and then every 3 weeks up to 9 weeks. Explants were evaluated for composition (double-stranded (ds)DNA, total collagen, sulfated proteoglycan, protein), osteogenic target gene mRNA expression (ALP, bone sialoprotein (BSP), osteocalcin (OCN), osteoprotegerin (OPG)), calcium and phosphorus content, and ultra- and microstructure. All materials and reagents were from Sigma-Aldrich, St. Louis, MO, USA, unless otherwise noted.

### Scaffold preparation (PEG/PLLA, GA; PEG/PLLA/TCP/HA, GT)

Scaffolds were fabricated through thermally induced phase separation (TIPS) as previously described [38]. Key components included PLLA [(C3H6O3)n, molecular weight (MW) 100,000- 150,000 g/mole], PEG [H(OCH2CH2)nOH, MW 1,900-2,200 g/mole], unsintered nano-sized HA (MW = 502.31 g/mole), and unsintered nano-sized β-TCP (MW=310.18 g/mole). The phase composition of HA and β-TCP powders were determined with an X-ray diffractometer (XRD; X'Pert PRO, PANalytical Co., Netherlands) employing Cu-Kα radiation (45kV, 40mA). Data were collected from 10^o^ to 90^o^ for 2Θ with a step size of 0.026^o^. 

#### PEG/PLLA (60:40)

Solutions of 10% PEG and 10% PLLA in 1,4-dioxane (AcroSeal™, ACROS Organics) were combined at a ratio of 6:4 (v/v) with stirring at 85 °C to create a homogenous mixture.

#### PEG/PLLA/TCP/HA (36:24:24:16)

TCP was added to 10% PEG at a ratio of 4:6 (TCP:PEG, w/w) and HA to 10% PLLA at a ratio of 4:6 (HA:PLLA, w/w) under the same conditions as above. Solutions were combined at a ratio of 6:4 (TCP-PEG:HA-PLLA), added to polydimethylsiloxane tubes (inner diameter 10 mm, depth 10 mm), and unidirectionally frozen by lowering the sample into liquid nitrogen from one end to provide a unidirectional porous structure. Samples were maintained at –80 °C overnight and subsequently lyophilized for 48 h to remove the solvent. Tubular scaffolds were sliced into discs (diameter 10 mm, depth 3 mm) that were individually packaged and sterilized with ethylene oxide at 57 °C for 2 h.

### Tissue harvest, cell expansion, cryopreservation

Sternal bone marrow and subcutaneous adipose tissue were harvested from seven thoroughbred geldings (6.3 ± 1.7 years of age; mean ± standard error of the mean (SEM)) as previously described [8]. Briefly, horses were sedated (detomidine HCl, 0.04 mg/kg intravenously (IV)), the sternebrae aseptically prepared, and 6 ml of 2% lidocaine chloride infiltrated into the soft tissues. With 14 G Jamshide needles, bone marrow was aspirated into heparinized syringes (1000 IU/60 ml bone marrow aspirate). Next, skin and subcutaneous tissues dorsolateral to the base of the tail were infiltrated with 2% lidocaine chloride. Skin was incised for 10 cm parallel to and approximately 15 cm lateral to the dorsal sacrum, and exposed subcutaneous adipose tissue (15–20 ml) was sharply excised. The skin was apposed with #2 nylon.

Cells were isolated as previously published with minor modifications [8]. Bone marrow aspirate combined with an equal volume of stromal medium (Dulbecco’s modified Eagle’s medium F-12 (DMEM/F-12, Hyclone, Logan, UT, USA), 1% antibiotic/antimycotic solution (MP Biomedical, Irvine, CA, USA), 10% fetal bovine serum (FBS; Hyclone)) was separated via centrifugation (350 × g, 4 °C, 30 min) with Ficoll-Paque® PLUS (Stem Cell Technologies, Vancouver, Canada). The cell layer was removed and centrifuged (260 × g, 4 °C, 5 min) to form a cell pellet that was seeded in T75 flasks (5 × 10^3^ cells/cm^2^) for culture. Stromal medium was refreshed after 24 h and then every 3 days. In this study, P0 is the first cell passage of primary cells. Procedures performed at temperatures other than room temperature are indicated.

Minced adipose tissue was combined with an equal volume of phosphate-buffered saline (PBS; Hyclone). After the mixture separated into two phases over 5 min, the infranatant was digested in 1% bovine serum albumin (BSA; Fisher Bioreagents, Fair Lawn, NJ, USA) and 0.1% collagenase type I (Worthington Biochemical Corporation, Lakewood, NJ, USA) in DMEM/F-12 for 2 h at 37 °C. After filtering (100-μm nylon cell strainers; BD Falcon, Bedford, MA, USA) and centrifugation (260 × g, 5 min), cells in DMEM with 1% BSA were added to an equal volume of red blood cell lysis buffer (0.16 mol/L NH_4_Cl, 0.01 mol/L KHCO_3_, 0.01% ethylenediaminetetraacetic acid (EDTA)) for 5 min. The stromal vascular fraction (SVF) was collected after centrifugation (260 × g, 5 min) and cultured as above.

At 70% confluence, cells were detached with 0.05% trypsin (Hyclone), suspended in cryopreservation medium (80% FBS, 10% DMEM/F-12, 10% dimethyl sulfoxide (DMSO; Fisher Scientific, Fair Lawn, NJ, USA)) at 1–1.5 × 10^6^ cells/ml and aliquoted into 1.5-ml cryovials (Fisher Scientific). Cells were cooled to –80 °C at approximately –1 °C/min (CoolCell®, BioCision, Larkspur, CA, USA) overnight and then transferred to liquid nitrogen for 33–45 days. To thaw, cryovials were placed in a 37 °C water bath for 1–2 min followed by centrifugation (380 × g, 5 min), a PBS wash, another centrifugation (380 × g, 5 min), and then stromal medium culture.

### Immunophenotype: flow cytometry

Passage 3 revitalized ASCs and BMSCs from three equine donors were combined in equal proportions within cell type for immunophenotype detection. Aliquots of 1 × 10^5^ cells in 150 μl PBS were incubated for 30 min at room temperature with 200 μg/ml labeled or unlabeled antibody (CD34-PE, CD29, CD73-FITC, CD44-FITC, CD90-PE, CD105-PE) specific or validated cross-reactive for equine antigens (Table 1). Cells were rinsed with PBS, centrifuged (350 × g, 5 min), and fixed with 4% neutral buffered formalin. For CD29, cells were incubated with labeled anti-immunoglobulin (IgG-FITC) for 30 min, rinsed with PBS, centrifuged (350 × g, 5 min), and fixed with 4% neutral buffered formalin. Cell fluorescence was quantified using a FACSCalibur flow cytometer and Cell Quest Pro software (BD Biosciences, San Jose, CA, USA). Autofluorescence was determined on antibody-negative samples.

**Table 1:** Flow cytometry antibodies

Antibody	Label	Marker expression	Manufacturer	Origin species	Target species	Diluent
CD29	N/A	β1 integrin	BD Biosciences	Mouse	Canine	PBS
CD44	FITC	Cell-surface glycoprotein, hyaluronic acid receptor	eBioscience	Mouse	Canine	PBS
CD73	FITC	5’-Nucleotidase	eBioscience	Mouse	Human	PBS
CD90	PE	Thy-1, fibroblasts, MSC, HSC	eBioscience	Mouse	Canine	PBS
CD105	PE	Type 1 glycoprotein	eBioscience	Mouse	Human	PBS
CD34	PE	HSC	BD Biosciences	Mouse	Canine	PBS
IgG	FITC	N/A	Sigma	Rabbit	Mouse	PBS

*FITC* fluorescein isothiocyanate, *HSC* hematopoietic stem cell, *IgG* immunoglobulin, *MSC* multipotent stromal cell, *N/A* not applicable, *PBS* phosphate-buffered saline, *PE* phycoerythrin

**Table 2:** In-vivo study design

Scaffold	Cell component			Analyses for individual mouse implants (6 per mouse)			
	None	BMSC	ASC	Gene expression	Composition	Ultrastructure	Microstructure
HT 40:60 β tricalcium phosphate:hydroxyapatite	7 mice	7 mice	7 mice	2/6 implants	2/6 implants	1/6 implants	1/6 implants
GA 60:40 polyethylene glycol:poly-l-lactic acid	7 mice	7 mice	7 mice	2/6 implants	2/6 implants	1/6 implants	1/6 implants
GT 36:24:24:16 polyethylene glycol:poly-l-lactic acid: β tricalcium phosphate:hydroxyapatite	7 mice	7 mice	7 mice	2/6 implants	2/6 implants	1/6 implants	1/6 implants

### Construct seeding and culture

**Fig. 1:**
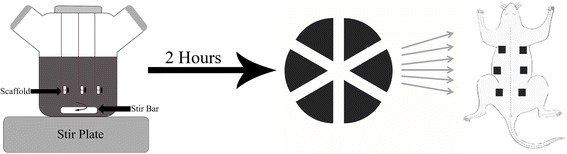
Schematic of spinner flask bioreactor cell loading, scaffold division, and implantation

P1 revitalized ASCs and BMSCs were culture expanded to P3 and then loaded onto scaffolds (1 × 10^6^ cells/scaffold) for 2 h with 70 rpm stirring in spinner flask bioreactors (37 °C, 5% CO_2_). Spinner flasks consisted of 100-ml flasks (Bellco® Biotechnology, Newark, NJ, USA) containing 120 ml of serum-free stromal medium and three separate 4-inch-long, 22-gauge spinal needles suspended from a rubber stopper at the top of each flask that each passed through the center of one scaffold (Fig. 1). Individual loading processes for scaffolds without cells, pooled aliquots identical to those used for immunophenotype, and for each cell tissue source and donor included one scaffold of each composition situated at the middle of the fluid. Specifically, there was one scaffold per donor (individual (7), pooled (2)/tissue source (BMSC, ASC, none)/composition (HT, GA, GT)) for a total of 81 samples. After 2 h, loading efficiency was determined and cell-scaffold constructs divided into six equal pieces for immediate evaluation, culture in stromal medium, or implantation as described below.

### Cell number via 3-(4,5-dimethylthiazol-2-yl)-2,5-diphenyl tetrazolium bromide (MTT)

Commercially available MTT (Cell Proliferation Kit I) was used to determine cell number immediately after cell loading or following 7 or 21 days of stromal medium culture in 24-well culture plates (two pooled isolates from three donors/cell tissue source/scaffold composition divided into six pieces for four replicates per time point). Briefly, constructs were gently rinsed with PBS and placed into fresh plates followed by incubation with 500 μl of a 5:1 mixture of stromal medium and MTT solution (5 mg/ml in PBS) for 2 h (37 °C, 5% CO_2_). Subsequently, 500 μl of DMSO was added to each well, the absorbance read at 540 nm (Synergy HT, BioTek Instruments, Winooski, VT, USA), and the cell number determined from equine ASC or BMSC standard curves. Cell number fold-change was calculated as C_f_/C_i_ (C_f_ = cell number after 7 or 21 days of culture; C_i_ = cell number immediately after scaffold loading).

### Scaffold surgical implantation

One scaffold divided into six pieces for each donor (7)/tissue source (BMSC, ASC, none)/composition (GA, GT, HT) was surgically implanted in the dorsal subcutaneous tissues of 63 male athymic mice (nu/nu, Charles River Laboratories, Wilmington, MA, USA) (Table 2). Implants were harvested 9 weeks after surgery and evaluated. Implants from each mouse were assessed for gene expression (*n* = 2 implants/mouse), composition (*n* = 2 implants/mouse), ultrastructure (*n* = 1 implant/mouse), and microstructure (*n* = 1 implant/mouse).

Mice were premedicated (glycopyrrolate, 0.02 mg/kg; butorphanol, 0.5 mg/kg, both subcutaneously) and anesthetized with isoflurane on oxygen delivered via a Baine circuit and mask. Following aseptic preparation, six 5-mm skin incisions were created equidistantly along the dorsum extending from the scapula to the sacrum approximately 1 cm ventral to each side of the spine. Following minimal blunt dissection, scaffolds were placed beneath the skin, subcutaneous tissues closed with #3-0 nylon, and the skin apposed with tissue glue.

### Radiographs: mineral deposition

Radiographs (Sound Technologies, Carlsbad, CA, USA) were performed immediately and 3, 6, and 9 weeks after surgery with mice in ventral recumbency and anesthetized as described above. Mineral deposition was subjectively assessed based on changes in radiopacity.

### Microcomputed tomography: porosity, bone volume/total volume

Two- and three-dimensional images generated from a microcomputed tomography (μCT) scanner (μCT 40; Scanco Medical AG, Bassersdorf, Switzerland) and graphics software (Mimics®, Materialise, Ann Arbor, MI, USA) were used to quantify specimen percent porosity and bone volume/total volume (BV/TV; μCT Evaluation Program V6.6), respectively, 9 weeks after implantation. Imaging technique (55 kV, 145 μA) and two-dimensional 16-bit grayscale threshold were identical among samples. Percent porosity was measured on slices at 25, 50, and 75% of the total specimen height. The mean of the three values was used as the measure for each specimen. To distinguish between high-contrast scaffold versus low-contrast newly deposited tissue, two thresholds were used for three-dimensional image reconstructions of GT (170, 370 mg HA/cm^3^) and HT (380, 590 mg HA/cm^3^) samples. The difference in the BV/TV between thresholds was considered as a measure of new tissue deposition. A single threshold (270 mg HA/cm^3^) was used for GA constructs which did not contain high-contrast material.

### Compositional analysis: DNA, hydroxyproline, sulfated glycosaminoglycan, protein

Compositional analysis was performed as per previous reports [39]. Briefly, constructs were lyophilized at –55 °C and 0.2 mbar for 4–5 h and then 20 mg of each sample was digested (9 mM di-sodium EDTA, 20 mM sodium acetate, 20 mM l-cysteine (MP Biomedicals, Solon, OH, USA), 2 mg papain (MP Biomedicals) per gram lyophilized sample) at 60 °C for 10 h [40, 41]. Digested samples were vigorously vortexed and then centrifuged (4000 × g, 10 min). Supernatants were stored at –80 °C until analysis. Double-stranded DNA was determined with a commercially available kit (Quant-iT™ PicoGreen® Kit, Invitrogen, Carlsbad, CA, USA) [41]. Hydroxyproline content as a measure of total collagen was quantified via Ehrlich’s colorimetric assay and absorbance at 550 nm based on trans-4-hydroxy-l-proline (ACROS Organics™, Morris Plains, NJ, USA) standards [41, 42]. Sulfated glycosaminoglycan (sGAG) was quantified with a dimethylmethylene blue (DMMB) assay, with absorbance at 520 nm and a chondroitin sulfate standard curve [41, 43]. Lowry’s total protein assay with Biuret’s and Folin-Ciocalteu’s reagents, absorbance at 650 nm, and bovine serum albumin standards, was used to quantify sample protein [44].

### Scanning electron microscopy: ultrastructure, phosphate, calcium

Samples were fixed with 2.5% glutaraldehyde in 0.1 M sodium cacodylate buffer (pH 7.4), post-fixed in 0.1% osmium tetroxide, and then dehydrated in a series of ethanol-distilled water solutions [8]. After critical point drying, they were sputter coated with gold and imaged (FEI Quanta 200, Netherlands). Surface calcium and phosphorus was measured in the center of each sample by energy-dispersive x-ray spectroscopy (EDS; 20 kV, 3% accuracy) [45].

### Real-time polymerase chain reaction (RT-PCR): gene expression

Total RNA was isolated from harvested samples (RNeasy Plus Mini Kit, Qiagen, GmbH, Germany), the concentration was determined spectrophotometrically (NanoDrop ND-1000; NanoDrop Technologies, Wilmington, DE, USA), and cDNA synthesized (QuantiTect Reverse Transcription Kit, Qiagen). Equine and murine osteogenic target gene levels (ALP, OCN, BSP, and OPG; Table 3) were quantified with RT-PCR using SYBR Green (Qiagen) technology and an MJ Research Chromo 4 Detector (Bio-Rad Laboratories, Hercules, CA, USA). Primers were designed according to species-specific genetic sequences. Each primer was tested with bone RNA to confirm the lack of cross-reactivity. The ΔCt values were determined relative to the reference gene glyceraldehyde 3-phosphate dehydrogenase (GAPDH).

**Table 3:** Primer sequences

Species	Lineage	Primer	Sequence	Accession number
Equine	Reference	GAPDH	Forward: AAGAAGGTGGTGAAGCAGGReverse: CTCAGTGTAGCCCAGGATG	NM_001163856.1
	Osteogenesis	ALP	Forward: GGAGTATGAGATGGACGAGReverse: GTAGTGAGAGTGCTTGTGCC	XM_005607380.2
		OCN	Forward: TGGCCCTGACTGCATTCTGReverse: CCCTCCTGCTTGGACATGAA	XM_005610022.1
		OPG	Forward: CCCCCCTTGTCCTGACCACTReverse: CGCCCTTCCTCACATTCG	XM_001916099.4
		BSP	Forward: GAAGAATCGGACGCTGAGReverse: ATCGTAGACAGGGTGGTG	XM_001496125.4
Murine	Reference	GAPDH	Forward: ACCCAGAAGACTGTGGATGGReverse: ACACATTGGGGGTAGGAACA	XM_017321385.1
	Osteogenesis	ALP	Forward: TGATGTGGAATACGAACTGGReverse: TGGGAATGCTTGTGTCTG	XM_006538500.2
		OCN	Forward: CCATCTTTCTGCTCACTCTGReverse: TTATTGCCCTCCTGCTTG	NM_007541.3
		OPG	Forward: CACTCGAACCTCACCACAGAReverse: GCTCGATTTGCAGGTCTTTC	NM_008764.3
		BSP	Forward: CAGCCATGAGTCAAGTCAGCReverse: CTTGTGGCTCTGATGTTCCA	NM_001204203.1

*ALP* alkaline phosphatase, *BSP* bone sialoprotein, *GAPDH* glyceraldehyde 3-phosphate dehydrogenase, *OCN* osteocalcin, *OPG* osteoprotegerin

### Light microscopy: microstructure

Following fixation in 4% neutral buffered formalin, serial sections (5 μm) of paraffin-embedded specimens were stained with Masson’s trichrome. Digital images were generated of all specimens (Leica DM 4500B, Allendale, NJ, USA).

### Statistical analysis

Statistical analyses were performed with the JMP statistical package (v13.0.0, SAS Institute, Cary, NC, USA). Mixed analysis of variance (ANOVA) models were used to evaluate cell number fold-change, composition (dsDNA, total collagen, sulfated proteoglycan, protein), gene expression, porosity, and BV/TV among scaffold compositions within cell tissue sources and between cell tissue sources within scaffold compositions. Fixed effects included scaffold composition and cell tissue source; equine donor and mouse were random effects. Tukey’s post-hoc tests were applied for multiple group comparisons (*p* < 0.05 was considered significant).

## Results

### Immunophenotype: flow cytometry

**Fig. 2:**
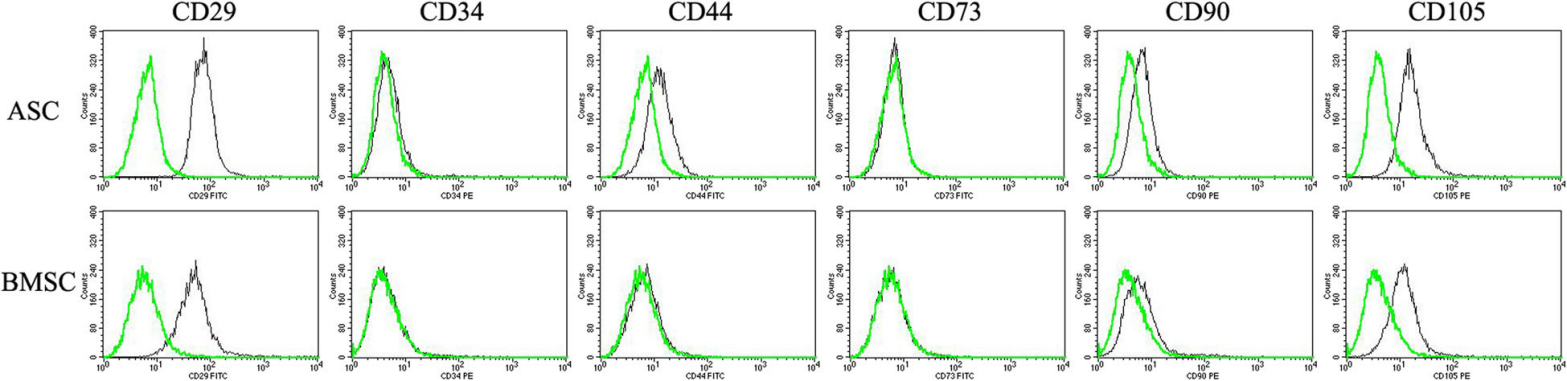
Immunophenotypes of P3 equine adipose-derived multipotent stromal cells (ASCs) and bone marrow-derived multipotent stromal cells (BMSCs) after culture expansion post-cryopreservation. The black lines represent labeled cells and the green lines represent autofluorescence

The majority of P1 ASCs and BMSCs were CD29^+^, CD105^+^, CD34^–^, CD73^–^ (Fig. 2). There were higher percentages of C90^+^, CD105^+^, CD44^+^ ASCs (Table 4).

**Table 4:** Percentages of CD29^+^, CD44^+^, CD90^+^, CD105^+^, CD34^–^, CD73^–^ P3 equine ASCs and BMSCs after post-cryopreservation culture expansion

	CD29^+^	CD34^–^	CD44^+^	CD73^–^	CD90^+^	CD105^+^
ASC	99	83.5	11.0	99.8	46.2	87.7
BMSC	90.3	97.9	1.5	99.9	28.2	58.8

*ASC* adipose-derived multipotent stromal cell, *BMSC* bone marrow-derived multipotent stromal cell

### Cell number: MTT

**Fig. 3:**
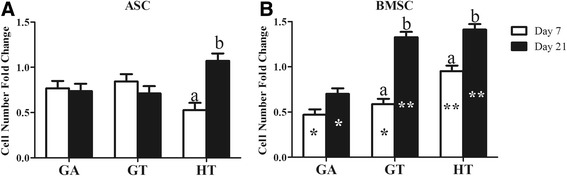
Fold-change in adipose-derived multipotent stromal cell (ASC; **a**) or bone marrow-derived multipotent stromal cell (BMSC; **b**) number after 7 or 21 days of static culture in stromal medium on scaffolds composed of tricalcium phosphate (TCP)/hydroxyapatite (HA) (40:60; HT), polyethylene glycol (PEG)/poly-l-lactic acid (PLLA) (60:40; GA), or PEG/PLLA/TCP/HA (36:24:24:16; GT). Columns with distinct superscripts are significantly different between culture times within scaffold composition and those with different asterisk (*) numbers are significantly different among scaffold compositions within culture time (*p* < 0.05)

The ASC and BMSC loading efficiencies were 81.2 ± 11.4% and 83.4 ± 4.4%, respectively. The ASC and BMSC number increased significantly in HT constructs between 7 and 21 days of culture, and BMSCs increased similarly in GT constructs (Fig. 3). After 7 days, the increase in BMSC number was greater in HT versus the other scaffolds, and after 21 days, the increase in GA was lower than the other two.

### Radiographs: mineral deposition

**Fig. 4:**
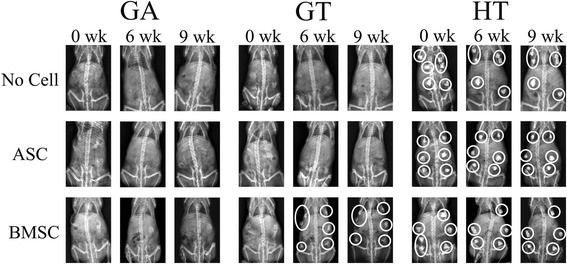
Radiographs of mice with carrier scaffolds composed of tricalcium phosphate (TCP)/hydroxyapatite (HA) (HT), polyethylene glycol (PEG)/poly-l-lactic acid (PLLA) (GA) or PEG/PLLA/TCP/HA (GT) with no cells or equine adipose-derived multipotent stromal cells (ASCs) or bone marrow-derived multipotent  stromal cells (BMSCs) 0, 6, and 9 weeks after surgical implantation. White circles surround radiopaque implants

All GA scaffolds and GT scaffolds with ASCs or without cells remained radiolucent throughout the study. Those GT scaffolds with BMSCs became radiopaque 6 weeks after implantation. Scaffolds composed of HT were radiopaque throughout the study, and scaffold margins became rounded with time (Fig. 4).

### μCT: porosity and BV/TV

**Fig. 5:**
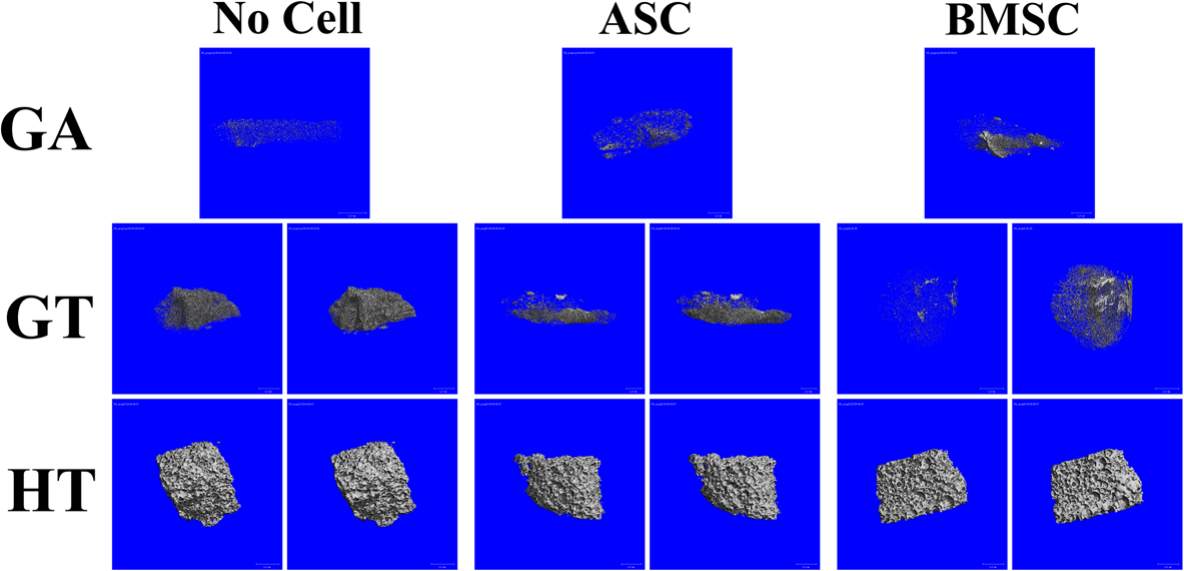
Three-dimensional explant reconstructions demonstrating models generated with high (left) and low (right) thresholds to distinguish between high contrast scaffold structure (left) versus low contrast newly deposited tissue (right). A single threshold was used for GA constructs due to the limited presence of high contrast material in the specimens. ASC adipose-derived multipotent stromal cell, BMSC bone marrow-derived multipotent stromal cell, GA polyethylene glycol (PEG)/poly-l-lactic acid (PLLA), GT PEG/PLLA/tricalcium phosphate (TCP)/hydroxyapatite (HA), HT TCP/HA

**Fig. 6:**
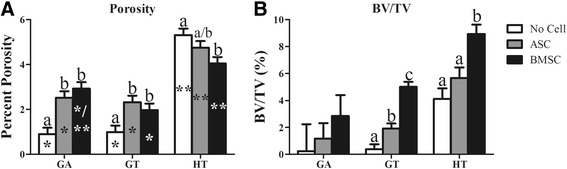
Percent porosity (**a**) and bone volume/total volume (BV/TV; **b**) (mean ± SEM) of equine ASC and BMSC constructs 9 weeks after subcutaneous implantation in a murine model. Columns with distinct superscripts are significantly different among cell tissue source within scaffolds and those with different asterisk (*) numbers are significantly different among scaffolds within cell tissue source (*p* < 0.05). ASC adipose-derived multipotent stromal cell, BMSC bone marrow-derived multipotent stromal cell, GA polyethylene glycol (PEG)/poly-l-lactic acid (PLLA), GT PEG/PLLA/tricalcium phosphate (TCP)/hydroxyapatite (HA), HT TCP/HA

There were measurable differences between BV/TV of GT and HT constructs at low and high thresholds, while BV/TV in GA constructs were only detectable at a high threshold (Fig. 5). The HT constructs had the highest percent porosity (Fig. 6a). Within scaffold compositions, GA and GT scaffolds with cells had higher porosity than those without, while HT scaffolds without cells had higher porosity than HT-BMSC constructs. Direct comparisons among scaffold BV/TV was not possible due to distinct thresholds used to create reconstructions. Within scaffold compositions, GT- and HT-BMSC constructs had the highest BV/TV, and GT-ASC constructs had higher BV/TV than those with no cells (Fig. 6b).

### Compositional analysis: DNA, hydroxyproline, sGAG, protein

**Fig. 7:**
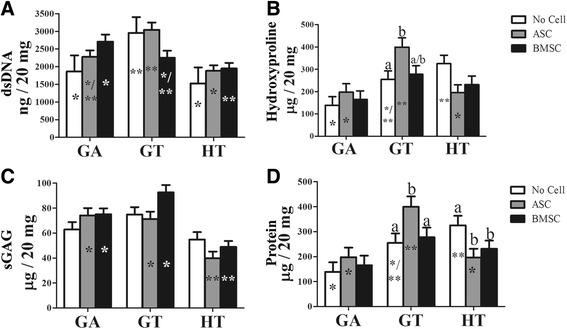
The double-stranded DNA (dsDNA; **a**), hydroxyproline (collagen; **b**), sulfated glycosaminoglycan (sGAG; **c**) and protein (**d**) content (mean ± SEM) in equine ASC and BMSC constructs 9 weeks after subcutaneous implantation in a murine model. Columns with distinct superscripts are significantly different among cell types within scaffolds and those with different asterisk (*) numbers are significantly different among scaffolds within cell types (*p* < 0.05). ASC adipose-derived multipotent stromal cell, BMSC bone marrow-derived multipotent stromal cell, GA polyethylene glycol (PEG)/poly-l-lactic acid (PLLA), GT PEG/PLLA/tricalcium phosphate (TCP)/hydroxyapatite (HA), HT TCP/HA

Significant differences in extracellular matrix (ECM) within scaffold composition included higher hydroxyproline (collagen) in GT-ASC constructs versus those without cells (Fig. 7b), higher protein in GT-ASC constructs versus no cells or BMSCs, and higher protein in HT scaffolds without cells versus either ASC or BMSC constructs (Fig. 7d). Differences among scaffolds within cell types included higher dsDNA in GT versus GA or HT scaffolds without cells, in GT versus HT scaffolds with ASCs, and in GA versus HT scaffolds with BMSCs (Fig. 7a). Hydroxyproline was higher in HT versus GA scaffolds without cells and GT versus GA or HT scaffold with ASCs (Fig. 7b). Both GA and GT scaffolds with ASCs or BMSCs had higher sGAG than HT with the same cells (Fig. 7c). Protein was higher in HT versus GA scaffolds without cells and GT versus GA or HT with ASCs (Fig. 7d).

### Scanning electron microscopy: ultrastructure, phosphate, calcium

**Fig. 8:**
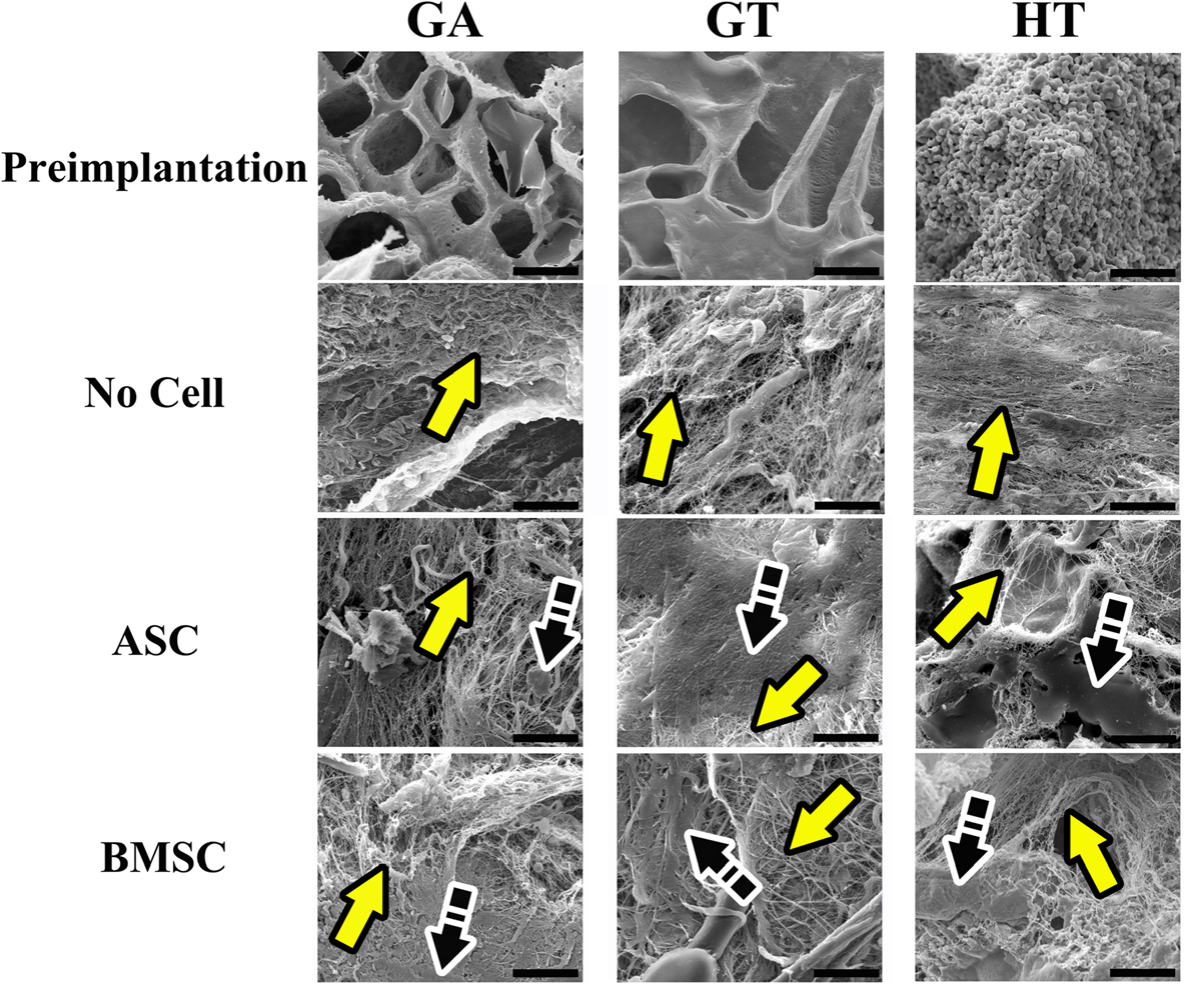
Scanning electron photomicrographs of scaffolds before cell loading (preimplantation) and 9 weeks after implantation without (no cell) or combined with equine ASCs or BMSCs. Magnification = 3000×; scale bar = 20 μm. Yellow arrows show collagen fibrils; black arrows show solid (mineralizing) region. ASC adipose-derived multipotent stromal cell, BMSC bone marrow-derived multipotent  stromal cell, GA polyethylene glycol (PEG)/poly-l-lactic acid (PLLA), GT PEG/PLLA/tricalcium phosphate (TCP)/hydroxyapatite (HA), HT TCP/HA

The XRD patterns of the HA and β-TCP were consistent with the manufacturer’s description with 19.3% and 6.3% crystallinity, respectively. Collagen fibrils and amorphous matrix were apparent in all explants (Fig. 8). A notable distinction was the presence of solid regions in various stages of mineralization and surrounded by randomly oriented collagen fibrils in scaffolds with cells. The well-delineated regions were distinct from the more proteinaceous ECM of scaffolds without cells. Subjectively, the BMSC construct ECM was the most dense among the explants.

**Fig. 9:**
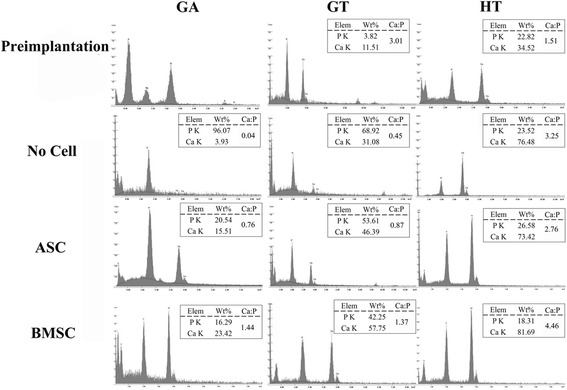
Energy dispersive x-ray microanalysis of explants before (preimplantation) cell loading or combined with equine ASCs or BMSCs 9 weeks after implantation. ASC adipose-derived multipotent stromal cell, BMSC bone marrow-derived multipotent stromal cell, GA polyethylene glycol (PEG)/poly-l-lactic acid (PLLA), GT PEG/PLLA/tricalcium phosphate (TCP)/hydroxyapatite (HA), HT TCP/HA

Within HT-BMSC and GA and GT constructs, calcium and phosphorus percentages were higher and lower, respectively, in scaffolds with versus without cells (Fig. 9), and ratios did not change appreciably in HT-ASC constructs. The calcium phosphorus ratio was closest to that of HA (1.6) in GA-BMSC and GT-BMSC constructs [46].

### RT-PCR: gene expression

#### Equine

**Fig. 10:**
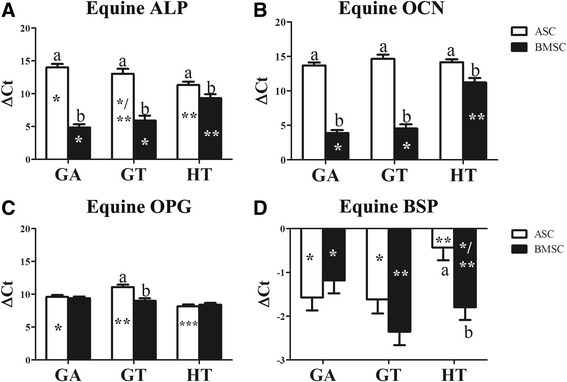
Equine alkaline phosphatase (ALP; **a**), osteocalcin (OCN; **b**), osteoprotegerin (OPG; **c**), and bone sialoprotein (BSP; **d**) levels (mean ± SEM) in equine MSC-scaffold constructs 9 weeks after implantation. Columns with distinct superscripts are significantly different between cell tissue source among scaffolds and those with different asterisk (*) numbers are significantly different among scaffolds between cell tissue source (*p* < 0.05). ASC adipose-derived multipotent stromal cell, BMSC bone marrow-derived multipotent stromal cell, GA polyethylene glycol (PEG)/poly-l-lactic acid (PLLA), GT PEG/PLLA/tricalcium phosphate (TCP)/hydroxyapatite (HA), HT TCP/HA

There was no detectable equine gene expression in scaffolds without cells. Osteogenic gene expression tended to be highest in scaffolds with BMSCs and varied among scaffold compositions. Within scaffold compositions, BMSC constructs had higher ALP and OCN than ASC constructs (Fig. 10a, b). The OPG and BSP expression was highest in GT- and HT-BMSC constructs, respectively (Fig. 10c, d). Within cell tissue source, ALP was higher in HT- versus GA-ASC constructs (Fig. 10a). Both ALP and OCN were lowest in HT-BMSC constructs (Fig. 10a, b). The OPG in GA-ASC constructs was higher and lower than GT- and HT-ASC constructs, respectively. The BSP was lower in HT- versus GA- and GT-ASC constructs. Additionally, GT-BMSC constructs had higher BSP than GA-BMSC constructs.

#### Murine

**Fig. 11:**
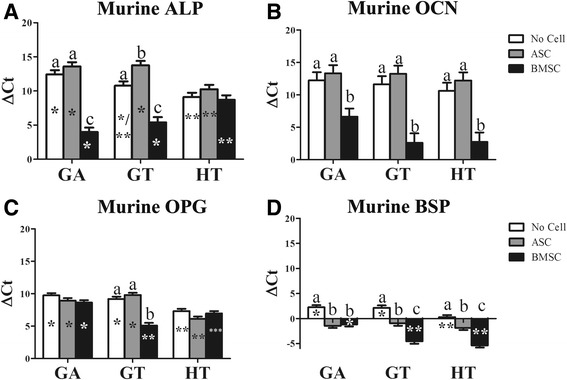
Murine alkaline phosphatase (ALP; **a**), osteocalcin (OCN; **b**), osteoprotegerin (OPG; **c**), and bone sialoprotein (BSP; **d**) levels (mean ± SEM) in equine MSC-scaffold constructs 9 weeks after implantation. Columns with distinct superscripts are significantly different between cell tissue source among scaffolds and those with different asterisk (*) numbers are significantly different among scaffolds between cell tissue source (*p* < 0.05). ASC adipose-derived multipotent stromal cell, BMSC bone marrow-derived multipotent stromal cell, GA polyethylene glycol (PEG)/poly-l-lactic acid (PLLA), GT PEG/PLLA/tricalcium phosphate (TCP)/hydroxyapatite (HA), HT TCP/HA

Osteogenic gene expression tended to be highest in scaffolds with BMSCs compared to those with no cells or ASCs. Differences among scaffold compositions varied and tended to parallel the equine gene expression. Among cell tissue source within scaffold composition, the highest expression levels included ALP in GA- and GT-BMSC constructs (Fig. 11a), OCN in all BMSC constructs (Fig. 11b), OPG in GT-BMSC constructs (Fig. 11c), and BSP in GT- and HT-BMSC constructs. Lowest expression levels included ALP expression in GT-ASC constructs (Fig. 11a) and BSP in all scaffolds without cells (Fig. 11d). Among scaffold compositions, ALP tended to be lower in GA and GT versus HT scaffolds with ASCs or without cells, and in HT versus GA and GT scaffolds with BMSCs (Fig. 11a). The OPG expression followed the same general pattern with the exception that it was lower in GA- versus HT-BMSC constructs (Fig. 11b). The BSP was highest in HT scaffolds without cells and lowest in GA-BMSC constructs.

### Light microscopy: microstructure

**Fig. 12:**
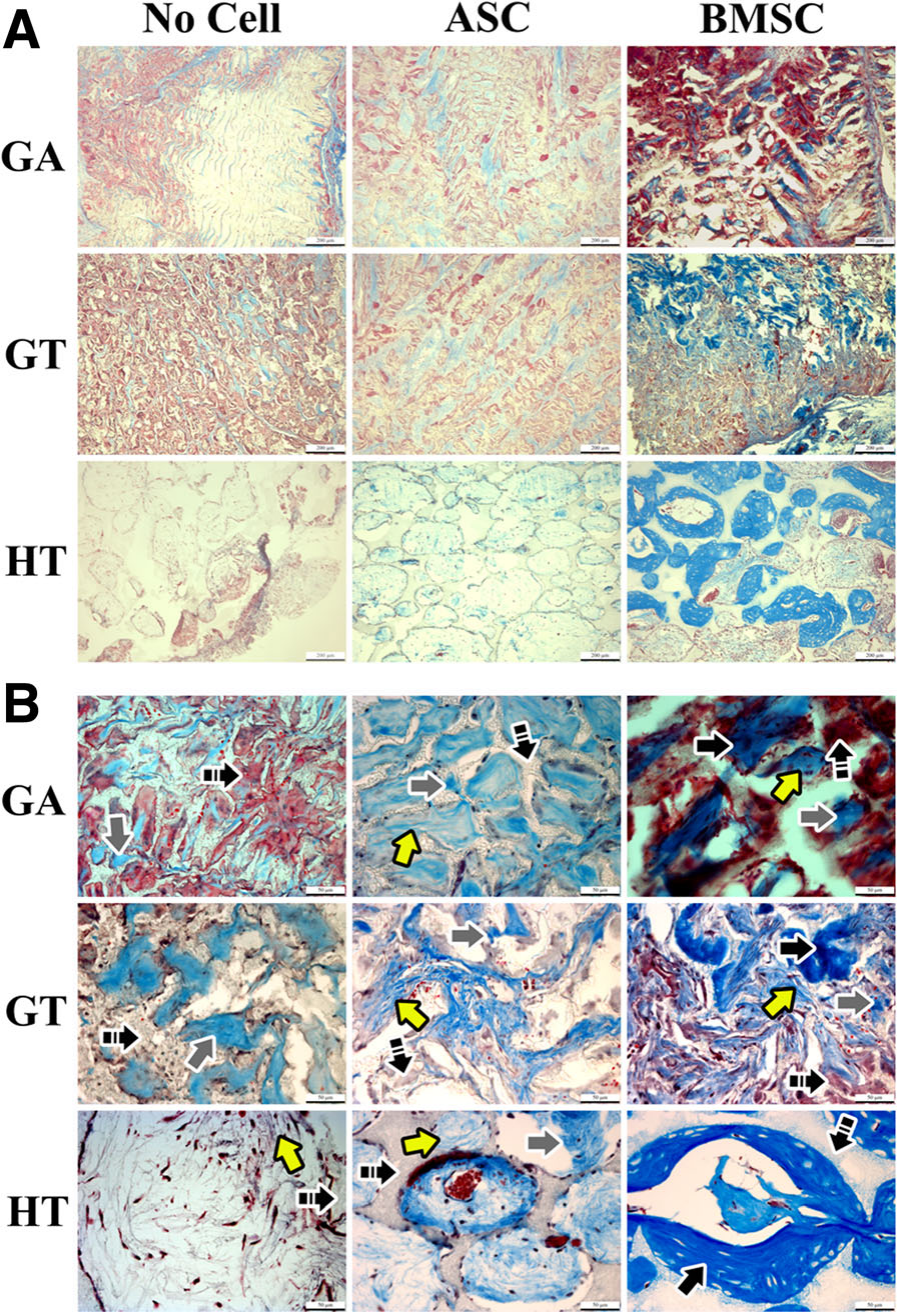
Light photomicrographs of equine MSC-scaffold explants 9 weeks after surgery. Masson’s trichrome stain. Magnification = 10×; scale bar = 200 μm (**a**); magnification = 40×; scale bar = 50 μm (**b**). Black arrows show osteoid, yellow arrows show collagen fibers; gray arrows show proteinaceous ECM; and two-stripe black arrows show scaffold. ASC adipose-derived multipotent stromal cell, BMSC bone marrow-derived multipotent stromal cell, GA polyethylene glycol (PEG)/poly-l-lactic acid (PLLA), GT PEG/PLLA/tricalcium phosphate (TCP)/hydroxyapatite (HA), HT TCP/HA

Regardless of composition, scaffolds with cells had more ECM than those without; most ECM deposition was on the implant periphery, and osteoid was apparent in all BMSC constructs. Specific to constructs, there was more amorphous, proteinaceous ECM apparent on GT- versus GA-ASC constructs, and GT-BMSC constructs had more osteoid ECM than GA-BMSC constructs (Fig. 12).

## Discussion

The overall conclusions from this study are that addition of mineral to polymer scaffolds enhances equine MSC osteogenesis over polymer alone, but mineral (HT) scaffolds provide superior support for equine MSC osteogenesis compared to either polymer composition. Parallel outcomes disprove both parts of the first hypothesis since osteogenesis was best on HT scaffolds regardless of cell tissue source, and BMSCs showed more robust osteogenesis than ASCs. Furthermore, GT scaffolds better supported osteogenic differentiation and ECM deposition than GA. However, the second hypothesis that the scaffolds with MSCs have greater osteogenesis than those without is true.

Equine fracture treatment can be more difficult than other species, in part due to their unique anatomy and obligatory quadrupedal gait and stance [47]. As in other species, autologous bone grafts are an established standard to augment and facilitate fracture healing [4]. The grafts vary in quality and quantity, and have harvest morbidity [48]. There are a number of synthetic graft options including cement, calcium phosphate based ceramics [49], polymers [20], bioglass [50], and metals [51], but few have been investigated for use in the horse, in part due to cost and inadequate mechanical properties [47]. Published studies evaluating the benefits of autologous equine MSCs to promote fracture healing have focused on clinical outcomes and stable fracture healing [52]. A challenge to clinical application of MSCs to the fracture site is a standardized, cost effective carrier scaffold [53]. The work in this series of investigations was designed to expand current knowledge base of potential graft materials to support equine MSC contributions to fracture healing. The results clearly demonstrate the potential for a scaffold composed of polymer and mineral to serve as bone graft material with and without MSCs. Polymer-mineral scaffolds may overcome some current limitations of available synthetic and autologous bone grafts and provide a standard mechanism for clinical application of MSCs for equine fracture therapy. This study also confirms the presence of exogenous mRNA in implants 9 weeks after placement. Most previous work confirms the persistence of implanted cells with inert cell labels or plasmid gene expression that can be incorporated into endogenous cells following exogenous cell senescence [54]. Few studies confirm the presence of exogenous mRNA to suggest, if not confirm, viable cell osteogenic gene expression. Another major finding is the largely parallel equine and murine genetic expression, including higher osteogenic gene expression by murine cells in BMSC constructs. Higher osteogenic potential of BMSCs compared to ASCs is consistent with established information and largely attributed to epigenetic factors [6, 55]. Observed upregulation of osteogenic gene expression by murine cells in equine BMSC constructs may be a consequence of both growth factor production, ECM deposition, and direct and indirect signaling by exogenous cells [56–60]. These findings are consistent with the knowledge of native cell recruitment and direction by MSC implants [9, 61], and they further emphasize the need to construct implants for optimum direction of both exo- and endogenous cell contributors to osteogenesis.

Study findings and current knowledge show that the polymer-mineral scaffold support of equine MSC osteogenesis will be improved with further optimization. Previous reports indicate that the addition of mineral to polymer scaffolds enhances MSC osteogenesis in other species, sometimes more than mineral alone [14, 45, 62]. A biomimetic environment is created, in part, by composition, surface topography, microarchitecture and mechanical properties [9]. Flexible polymer scaffolds with less structural organization in this study may have weaker osteogenic signaling than the rigid, porous ceramic structure. Calcium ions released from calcium phosphate-based minerals play a major role in influencing osteoblastic differentiation [63], and the presence of either HA or TCP enhances mRNA levels of BSP in human BMSCs without osteogenic medium [64]. Unsintered calcium phosphate with low crystallinity similar to that in this study enhance scaffold mechanical properties and contribute to human ASC osteogenic differentiation [38]. The crystals release calcium more quickly than sintered micro- or macroscale counterparts owing to more rapid dissolution [65]. Based on equine MSC osteogenic gene expression on scaffolds with calcium phosphate (HA and TCP) in this study and on collagen in previous work, polymer scaffolds with three-dimensional bone microstructure overlaid with minerals and collagen may better support osteogenesis [8, 66]. Scaffolds specifically designed for ASC osteogenesis could also possibly increase contributions to osteogenesis.

Higher ECM and osteoid deposition on the scaffold periphery may have resulted from cell distribution and in vivo conditions. Cell distribution was not confirmed, so cells may have concentrated on the scaffold periphery from spinner flask centrifugal forces and lack of continuous scaffold pores [67–70]. Better interstitial flow on the construct surface in vivo could have promoted better cell survival than in the center, despite small implant size [20, 30, 71]. While not directly translatable to in vivo, reduced in-vitro MSC numbers at 7 and 21 days compared to the initial number in half of the constructs may account for the lower in-vivo ECM deposition. The cause of the cell loss was not evaluated, but increased cell numbers in some scaffolds is inconsistent with scaffold cytotoxicity. The MSC affinity for culture plastic in vitro or surrounding tissues in vivo may have been equal or greater than that for scaffold components [72, 73]. Explant dsDNA content does not suggest cell loss is likely due to murine cells from the local environment in vivo. Future studies to evaluate cell distribution and orthotopic ECM deposition will help resolve these points.

The presence of equine MSCs significantly enhanced ECM deposition in scaffolds regardless of composition, although scanning electron microscopy and light microscopy confirmed osteoid deposition was highest with either BMSCs and/or high scaffold mineral content. Changes in EDS calcium and phosphate may indicate early ion deposition for mineralization [74]. Compositional analysis confirmed proteinaceous ECM in MSC constructs that was observed with light microscopy. A lower ECM composition in HT constructs is likely from ECM deposition throughout GT and GA scaffolds versus HT scaffold pore surfaces. The HT scaffold porosity may have decreased from ECM deposition within pores and polymer scaffold porosity increased from ECM deposition and organization.

The results of this study are limited to ectopic versus orthotopic ossification that is more representative of implant efficacy to support osteogenesis in native bone [75, 76]. Athymic mice have diminished osteogenic capabilities compared to immunocompetent animals [77], so scaffolds and cell-scaffold constructs may behave differently in an equine fracture. Nonetheless, subcutaneous implantation is an accepted approach with numerous intra-animal replicates to compare in vivo ECM deposition and genetic expression among scaffolds and cell-scaffold constructs [78, 79]. The study length exceeded the time for natural, early fracture stabilization [80] to evaluate long-term differences in cell-construct in vivo behaviors. Results that differ between this and other studies can be attributable to study duration, distinct in vivo and in vitro environments [81, 82], species [83, 84], and revitalized MSCs, a point that has been shown to impact cell behavior [85, 86] within immunophenotypes [87, 88]. Nonetheless, the number of sample replicates, duration, and parallel outcome measures in this study provide robust tests of the stated hypotheses.

The scaffolds used in the present study have been rarely studied in equine medicine due to the fact that bone graft study in veterinary medicine is far behind that in human medical research [81]. In equine medicine, the major bone graft sources are fresh, autogenous cancellous bone, and tissue engineered bone graft is a relatively new field. In our present study, this is the first time the polymeric-inorganic scaffold has been used to investigate a potential application in equine bone tissue engineering through in vitro and vivo evaluation. The presence of equine-specific mRNA confirm the contribution of equine MSCs after implantation. The addition of minerals to polymeric materials holds promising potential for an equine bone tissue engineering application. Additionally, the survival of implanted equine cells and the recruitment of murine MSCs provides strong support for MSC-based therapy for equine bone fracture treatment.

## Conclusions

These in-vivo study results confirm that addition of minerals to polymer carrier scaffold enhances strong osteogenic capabilities of equine MSCs without osteogenic preconditioning prior to implantation, and that BMSCs appear to have better osteogenic ECM production and organization than ASCs. Findings from these investigations confirm that contributions of both exogenous cells and recruitment of host MSCs are vital to MSC construct osteogenesis. The contributions in the present study may provide important information to help overcome equine bone healing challenges. The novel scaffold carriers evaluated in this study are appropriate for continued development of synthetic graft materials and viable tissue implants to augment equine fracture strategies.

## Abbreviations

μCT: Microcomputed tomography
ALP: Alkaline phosphatase
ASC: Adipose-derived multipotent stromal cell
BMSC: Bone marrow-derived multipotent stromal cell
BSA: Bovine serum albumin
BSP: Bone sialoprotein
BV/TV: Bone volume/total volume
DMEM/F-12: Dulbecco’s modified Eagle’s medium F-12
DMMB: Dimethylmethylene blue
DMSO: Dimethyl sulfoxide
ds: Double-stranded
ECM: Extracellular matrix
EDS: Energy-dispersive x-ray spectroscopy
EDTA: Ethylenediaminetetraacetic acid
FBS: Fetal bovine serum
GA: PEG/PLLA
GAPDH: Glyceraldehyde 3-phosphate dehydrogenase
GT: PEG/PLLA/TCP/HA
HA: Hydroxyapatite
HT: TCP/HA
IgG: Immunoglobulin
IV: Intravenously
MSC: Multipotent stromal cell
MTT: 3-(4,5-Dimethylthiazol-2-yl)-2,5-diphenyl tetrazolium bromide
MW: Molecular weight
OCN: Osteocalcin
OPG: Osteoprotegerin
P: Passage
PBS: Phosphate-buffered saline
PEG: Polyethylene glycol
PLLA: Poly-l-lactic acid
RT-PCR: Real-time polymerase chain reaction
SEM: Standard error of the mean
sGAG: Sulfated glycosaminoglycan
SVF: Stromal vascular fraction
TCP: Tricalcium phosphate
